# Mechanical conflicts at the tricuspid level. A rare cause of both atrial and ventricular lead damage

**DOI:** 10.1016/j.hrcr.2020.04.022

**Published:** 2020-05-08

**Authors:** Alex Scripcariu, Pierre Mondoly, Quentin Voglimacci-Stephanopoli, Franck Mandel, Philippe Maury

**Affiliations:** ∗ Department of Cardiology, University Hospital Rangueil, Toulouse, France; † Unité INSERM U 1048, Toulouse, France

**Keywords:** Insulation breaches, Lead fracture, Overdetection, Tricuspid valve, Ventricular systole

## Introduction

Insulation breaches of both atrial and ventricular leads at the site of the tricuspid valve are rarely observed. They may be caused by mechanical conflicts owing to repeated systolic valve closure or excessive slack and lead collision at this level.

## Case report

A 71-year-old man with nonischemic cardiomyopathy was referred for implantable cardioverter-defibrillator (ICD) shock during lunchtime, occurring without strenuous activity or ample upper body movements. This was preceded by multiple alarms identified by telecardiology. He was otherwise completely asymptomatic and had been implanted with a cardiac resynchronization therapy defibrillator device (Sorin Platinium; MicroPort, Clamart, France) 2 years earlier for dilated cardiomyopathy secondary to genetic mitochondrial disease, with left bundle branch block and reduced left ventricular ejection fraction (25%). Atrial lead was a bipolar Sorin PS55D SonRtip (MicroPort) with silicon elastomer insulation, while defibrillator lead was a single-coil Boston Endotak Reliance (Boston Scientific, Marlborough, MA) DF4 SG, also with silicon insulation.

**Figure 1:**
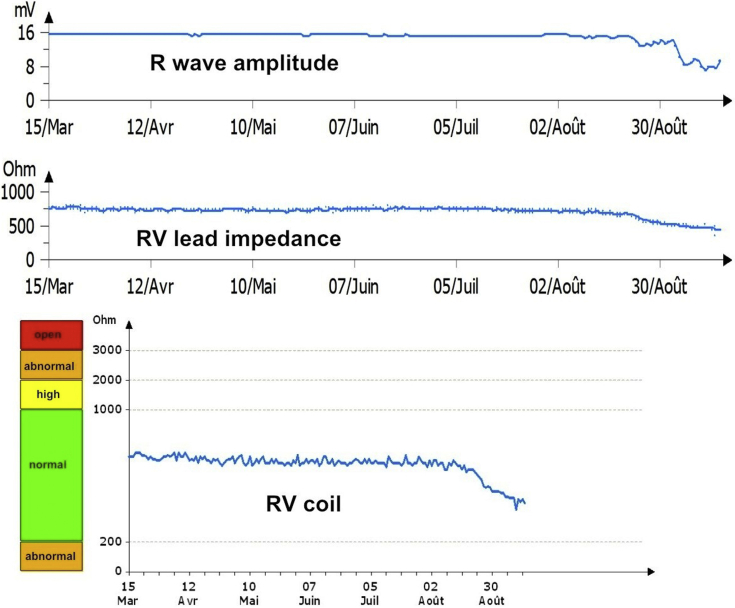
Implantable cardioverter-defibrillator logs showing evolution of R-wave amplitude and impedances of right ventricular (RV) lead and coil over time. In Sorin devices (MicroPort, Clamart, France), high circuit impedance is measured through the coil and the pacing electrodes, leading to higher normal values compared to other manufacturers.

Electrocardiography during normal sinus rhythm demonstrated unremarkable biventricular pacing. Device interrogation found a battery voltage of 2.97 V with an estimated life span of 7–9 years; an atrial lead impedance of 417 ohms; P-wave amplitude of 6.1 mV and atrial pacing threshold of 1.5 V; right and left ventricular lead impedances of 453 and 603 ohms, respectively; high-voltage circuit (coil) impedance of 460 ohms (normal range for Sorin devices); R wave of 6 mV; and right and left ventricular pacing thresholds < 1 V. Although remaining in the normal range, slight decreases in R-wave amplitude, right ventricular lead, and coil impedances were noted during the month prior to the event (Figure 1).

**Figure 2:**
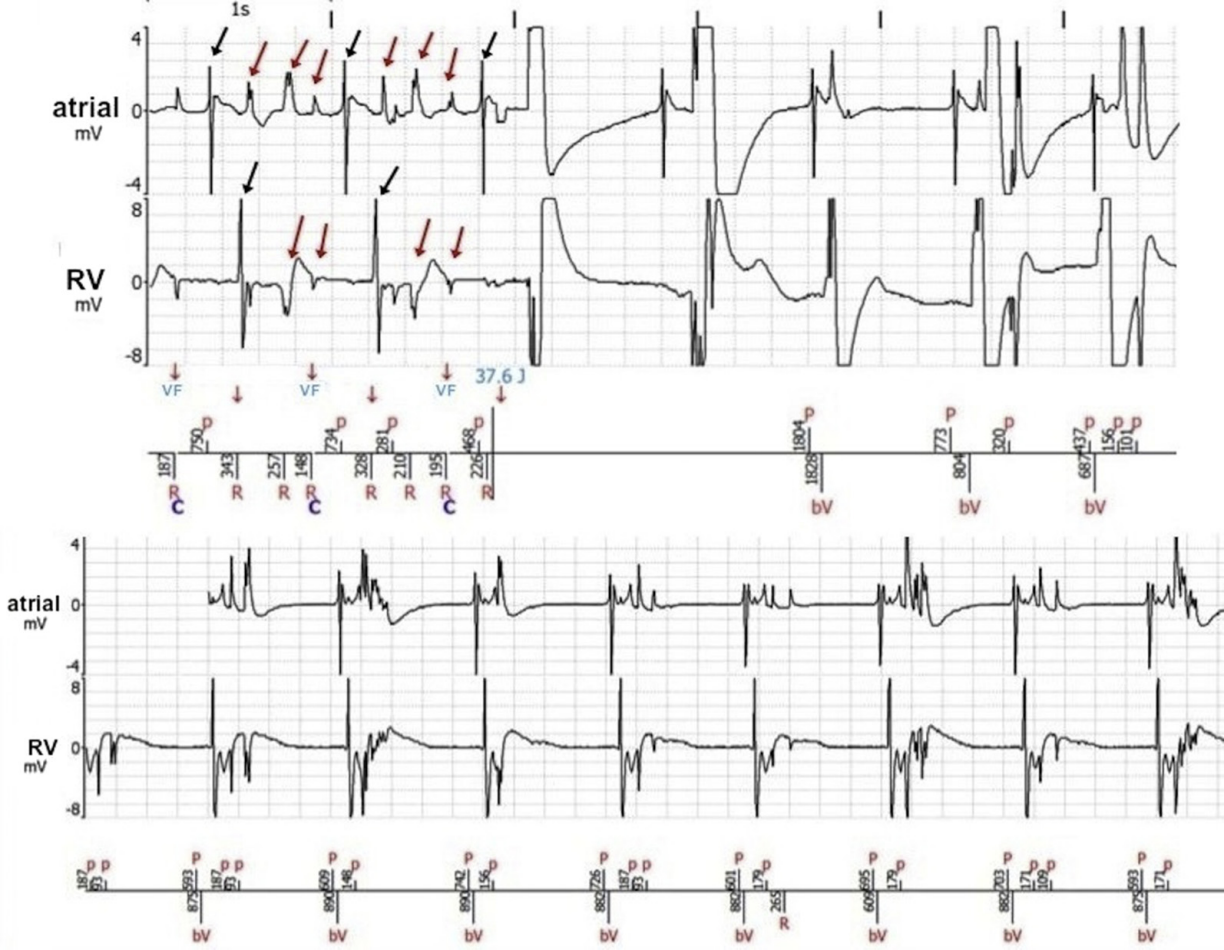
**Upper:** Both atrial and ventricular electrograms during the episode of “ventricular fibrillation” with occurrence of a 37.6 J implantable cardioverter-defibrillator shock. Black arrows indicate spontaneous atrial and ventricular events. Note the presence of nonphysiological artifact signals on both leads (*red arrows*) extending over the duration of mechanical ventricular systole (see text). **Lower:** Electrogram artifacts on atrial and right ventricular leads, as seen in telemedicine a few days prior to the event, leading to “mode switch.” As in upper panel, noise artifacts on both atrial and ventricular leads extend during ventricular systole. bV = biventricular paced event; C = condensator charge; P = atrial sensed event; p = atrial sensed event inside the refractory period; R = ventricular sensed event.

**Figure 3:**
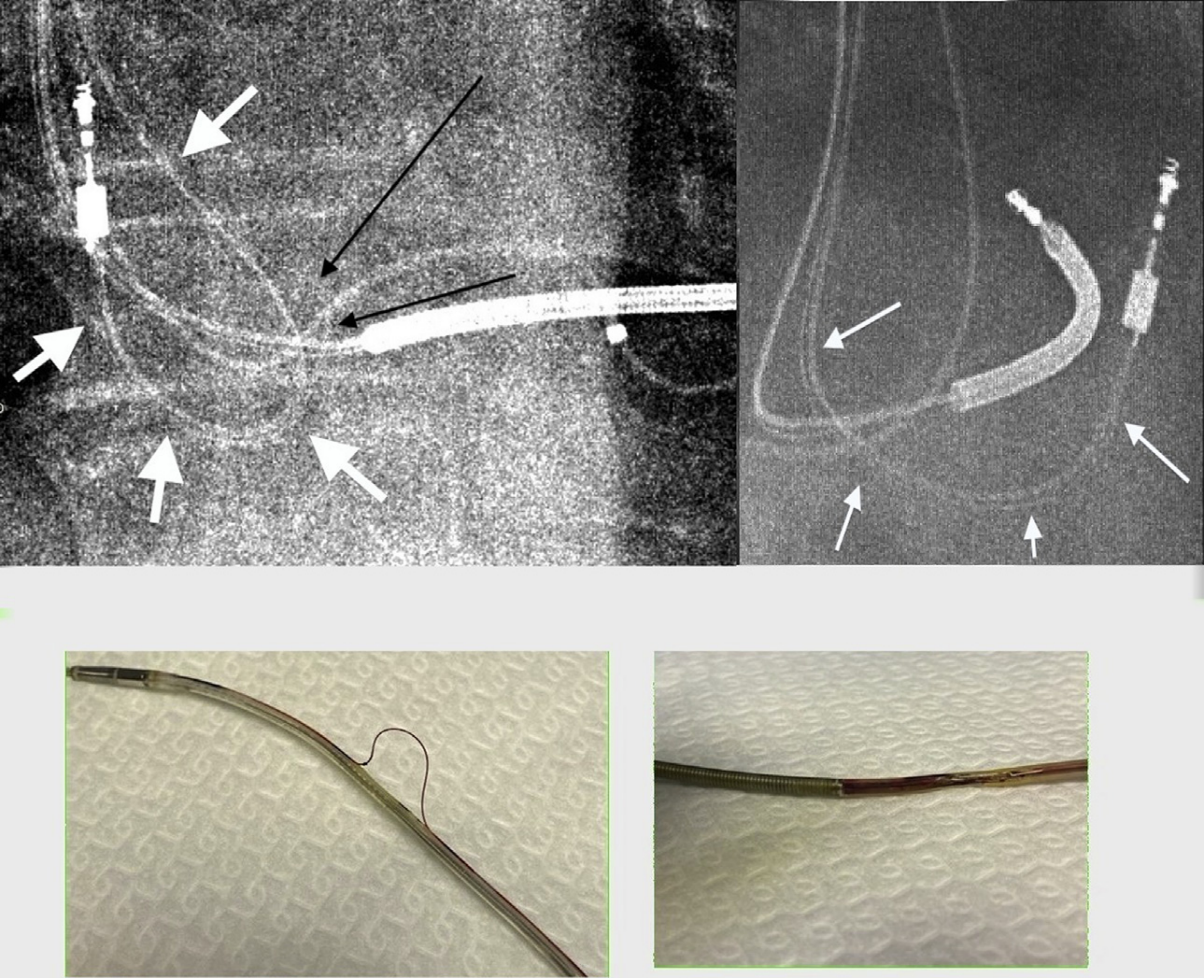
**Upper:** Anterior-posterior and lateral views of chest radiographs. Leads appear to be normally placed, although the atrial lead was making a loop, evoking excessive slack, descending very low in the right atrium probably neighboring the tricuspid valve (*white arrows*). Cautious inspection reveals conductor externalization (*black arrow*). **Lower:** Insulation breaches of atrial (left) and right ventricular (right) leads, with the atrial lead additionally showing an externalized conductor.

An episode classified as “ventricular fibrillation” treated by an internal ICD shock was found in ICD memory. Electrogram analysis confirmed that the shock was inappropriate, with additional counting of nonphysiological artifact signals on right ventricular but also on atrial leads (Figure 2), which pointed to a dual lead malfunction. Multiple previous atrial inappropriate detections were also seen in telemedicine a few days prior to the event (Figure 2), leading to repetitive alarms. This, together with decreased impedances on the right ventricular lead, evoked an insulation breach on both leads. Chest radiographs did not reveal leads abnormalities at first look, except a wide looping of the atrial lead in the low right atrium (Figure 3).

A decision was made to extract both leads and the device. Extraction of the atrial lead by a flexible sheath and of the ventricular lead by means of a snare from a femoral approach were performed. Postextraction lead examination showed insulation breaches of both atrial and ventricular leads (Figure 3) together with the externalized conductor on the atrial lead. The coating defect lay approximately 6 cm from the tip of the right ventricular lead and 5 cm from the tip of the atrial lead. Comparing to chest radiographs, this seemed to be the spot where the ventricular lead crossed the tricuspid annulus and to correspond to the excess slack regarding the atrial lead. There was no anomaly noted concerning the structure of the tricuspid valve on transthoracic echocardiography.

This was a case of lead-on-lead abrasion resulting in insulation defects on both leads. The most likely explanation is the incessant mechanical conflicts against the tricuspid valve, possibly related to systolic displacements/constraints/collisions on every ventricular systole and largely favored by an excessive slack on the atrial lead.

## Discussion

The overall current incidence of lead failure is 1.3 per 100 lead-years^1^ with an annual failure rate increasing progressively with time after implantation, reaching 20% at 10 years.^2^ Overall survival rates of ICD leads are 90% at 5 years, with a current incidence of 0.28% to 1.14% lead failure after exclusion of recalled leads.^3^ Most fractures occurred inside the pacemaker pocket, from the connector to the venous entry, often implying compression between the clavicle and the first rib (“subclavian crush syndrome”).^4^^,^^5^ Although blood vessels can be compressed by the clavicles, damage to leads in that region is rather known to be caused by soft tissue entrapment and repetitive movements rather than true bone contact.^6^

The tricuspid valve has already been reported as a potential unusual site of pacemaker lead fracture, but cases are scarce and without clear evidence of the mechanism.^4^ In our case, the location of both leads’ damages at the level of the tricuspid valve strongly evoked local mechanical constraints. This could have been caused by repeated systolic movements, torsions, and conflicts owing to every ventricular systole, which occurred roughly 80,000 times a day. The time of occurrence of artifact potentials seen on electrograms on both leads, ranging from after the QRS up to the expected end of the mechanical ventricular systole, is also highly evocative of this mechanism (Figure 2). Mechanical systole is expected to last approximately 400 ms for a ventricular cycle of 750 ms using the formula developed by Boudoulas and colleagues^5^ showing the correlation between mechanical systole and heart rate, although pacing probably still delays the end of ventricular systole. The atrial lead was possibly damaged in this case because of the excessive looping/slack neighboring the tricuspid valve, which may favor collisions against the ventricular lead and the tricuspid annulus after each ventricular systole as well. Thus, repetition of ventricular systole and lead collisions may alter lead insulation or conductor at the site of principal mechanical bending/torsion/collision (ie, the tricuspid annulus), further creating noise artifacts or overdetections of intracardiac events at this point in the cardiac cycle.

Since such mechanical conflict is only relevant in exceptional cases and may need years to translate into lead defects, it is difficult to propose a special monitoring other than conventional management. Chest radiographs are of limited interest for detecting lead defects. Implantation techniques may also be difficult to reconsider owing to the rarity of this complication, lack of alternative standard technique for transvenous leads, and challenges of avoiding mechanical conflict at the tricuspid level.

## Conclusion

Transvenous leads may be occasionally damaged at the tricuspid level when excessive slack, associated with incessant mechanical interaction owing to ventricular systole, results in lead-on-lead insulation abrasion. This case highlights the reality of this phenomenon, whose consequences, however, are exceptional. The development of leadless pacemakers and subcutaneous ICDs may render this complication even less relevant in the future.

**Key Teaching Points**

• The tricuspid valve is a potential but unusual site of pacemaker lead fracture.
• Insulation breaches of intracardiac leads at the site of the tricuspid valve may possibly be due to mechanical conflicts because of repeated systolic valve closure.
• Lead damages at the level of the tricuspid valve may provoke overdetection or electrical noise occurring only after the QRS.
• Excessive slack of atrial leads may create mechanical conflict with other leads.
